# Cerebral Perfusion in Chronic Stroke: Implications for Lesion-Symptom Mapping and Functional MRI

**DOI:** 10.3233/BEN-2011-0283

**Published:** 2011-05-23

**Authors:** Jessica D. Richardson, Julie M. Baker, Paul S. Morgan, Chris Rorden, L. Bonilha, Julius Fridriksson

**Affiliations:** ^1^Department of Communication Sciences and DisordersUniversity of South CarolinaColumbiaSCUSA; ^2^Department of Radiology and Radiological ScienceMedical University of South CarolinaColumbiaSCUSA; ^3^Georgia State/Georgia Tech Center for Advanced Brain ImagingGAUSA; ^4^Department of NeurosciencesMedical University of South CarolinaColumbiaSCUSA

**Keywords:** Aphasia, arterial spin labeling (ASL), hypoperfusion, ischemic stroke, peri-infarct

## Abstract

Lesion-symptom mapping studies are based upon the assumption that behavioral impairments are directly related to structural brain damage. Given what is known about the relationship between perfusion deficits and impairment in acute stroke, attributing specific behavioral impairments to localized brain damage leaves much room for speculation, as impairments could also reflect abnormal neurovascular function in brain regions that appear structurally intact on traditional CT and MRI scans. Compared to acute stroke, the understanding of cerebral perfusion in chronic stroke is far less clear. Utilizing arterial spin labeling (ASL) MRI, we examined perfusion in 17 patients with chronic left hemisphere stroke. The results revealed a decrease in left hemisphere perfusion, primarily in peri-infarct tissue. There was also a strong relationship between increased infarct size and decreased perfusion. These findings have implications for lesion-symptom mapping studies as well as research that relies on functional MRI to study chronic stroke.

## 1. Introduction

Lesion symptom mapping studies attempt to infer brain function by associating the behavioral deficits observed with the location of injury observed using brain imaging. Much has been learned about brain-behavior relationships from lesion-symptom mapping studies. However, a crucial assumption of such studies is that behavioral impairments reflect frank tissue damage and that neurophysiology is normal in the remaining brain areas that appear structurally intact. Although impaired cerebral perfusion is commonly reported in the acute and sub-acute phases of stroke (see [3,7,11,13,22]), a handful of studies have reported abnormal perfusion in chronic stroke [1,5,12,19]. It is generally believed that hypoperfusion resolves over time, in accordance with early behavioral recovery patterns [3] and if present, is probably functionally insignificant [13,21]. However, several single case studies of chronic stroke report hypoperfusion in the hemisphere that incurred the damage [12,13,19] and carefully relate it to functional deficits [13,19], complementing an earlier study reporting the "occasional phenomenon" of hypoperfusion in chronic stroke [1].

Based on the findings reported above, it seems that attributing specific behavioral impairments to localized brain damage in chronic stroke leaves much room for speculation, as the impairments could also reflect abnormal function in structurally intact brain regions. If

ISSN 0953-4180/11/$27.50 © 2011 – IOS Press and the authors. All rights reserved compromised cerebral perfusion is common in chronic stroke, it may need to be taken into account when examining lesion-impairment relationships. Unfortunately, research focused on cerebral perfusion in chronic stroke is limited, and little is known about perfusion deficits in remaining brain areas or how far such deficits might extend beyond the infarct. To shed light on this issue, the present study provides preliminary information regarding the relationship between cerebral perfusion, time post onset (TPO) of stroke, and infarct size in chronic patients with cortical stroke.

## 2. Materials and methods

### 2.1. Patients

The patients examined in this research were a subset of a larger group consecutively enrolled for an ongoing aphasia treatment study. The 17 participants (10 females; age range = 41–81 years; $M = 61.82$ years) had each incurred a single cortical ischemic stroke of the left hemisphere at least 4 months prior to study inclusion ($M = 48.47$ months, range = 4–246 months). The presumed etiology of stroke was defined by interviewing patients regarding their stroke risk factors. Two patients had been diagnosed with atrial fibrillation at the time of the stroke, and two patients were diagnosed with symptomatic extracranial anterior circulation stenosis. The other patients were classified as having suffered cryptogenic or small vessel strokes. The study was approved by the University of South Carolina's Institutional Review Board, and informed consent was obtained from all patients.

### 2.2. Imaging

All MRI data were collected using a 3T Trio system (Siemens Medical, Erlangen, Germany) with a 12-element head coil. Cerebral perfusion was estimated using a Pulsed Arterial Spin Labeling (PASL) sequence [25]. PASL imaging utilized a single shot gradient echo planar imaging (EPI) sequence with the following parameters: parallel imaging (GRAPPA) factor = 2, no fat suppression, matrix size = 64 × 64 mm, pixel size = 3.5 × 3.5 mm, 16 axial slices (5 mm thick with 6 mm center-center slice separation), TR = 4000 ms, TE = 12 ms, and bandwidth = 3 kHz/pixel. The selective inversion slab was 120 mm thick; the inferior saturation slab was 100 mm thick and directly below the imaging slices. The post inversion delay was 700 ms and the post labeling delay was 1000 ms. Thirty repeats of selective/non-selective image pairs were acquired. An estimate of the equilibrium magnetization (M0) was obtained by a further acquisition of two repeats of the same sequence (but TR = 10 s and post inversion delay = 5 s). Images were corrected for head motion and mean images of the selective and non-selective images constructed. Perfusion images were processed using in-house software (www.nottingham.ac.uk/∼njzwww/paul/software/perf.html), and values were calculated in units of ml/min/100g tissue, as described previously [25].

To improve coregistration of images and infarct demarcation, all participants were scanned with high-resolution T2 and T1 MRI sequences described previously [10]. Structural images were prepared for data analysis using software designed and supported by the Oxford Centre for Functional MRI of the Brain (FMRIB) – FMRIB's Software Library (FSL) version 4.1 [24]. Cropped and skull-stripped structural MRI images were normalized to standard space, employing infarct weighting for improved accuracy. Normalized images were resliced to 2 mm isotropic. Each participant's perfusion images were then coregistered to their own spatially normalized structural images.

Infarcts were demarcated on axial slices of the normalized T2 MRI using MRIcron [23], with the high-resolution native T2 MRI used to guide demarcation. To create the peri-infarct mask, the infarct was dilated 8 mm, and the original infarct was subtracted from the diameter of the dilated infarct. Also subtracted was the peri-infarct area between 0 to 3 mm beyond the infarct's rim (partial-volume boundary) in order to account for partial lesion volume. This was overlaid on a probabilistic gray matter mask, producing the left hemisphere peri-infarct gray matter (LHp) mask. We also created a mask for the remaining left hemisphere (LHr) by beginning with the probabilistic gray matter mask for the left hemisphere and removing all regions that were part of the LHp, infarcted tissue, or infarct-LHp partial-volume boundary. The masks were flipped to obtain the right hemisphere peri-infarct gray homologue (RHp) and right hemisphere remaining gray homologue (RHr) masks (see Fig. 1). These four volumes of interest (VOIs) were overlaid on the perfusion images; mean CBF values were obtained for each utilizing MRIcron. In addition, relative values were obtained by expressing each VOI value as a percentage of its mirror VOI (i.e., perfusion ratio).

### 2.3. Data analyses

Statistical analyses were performed using SPSS (Sta-

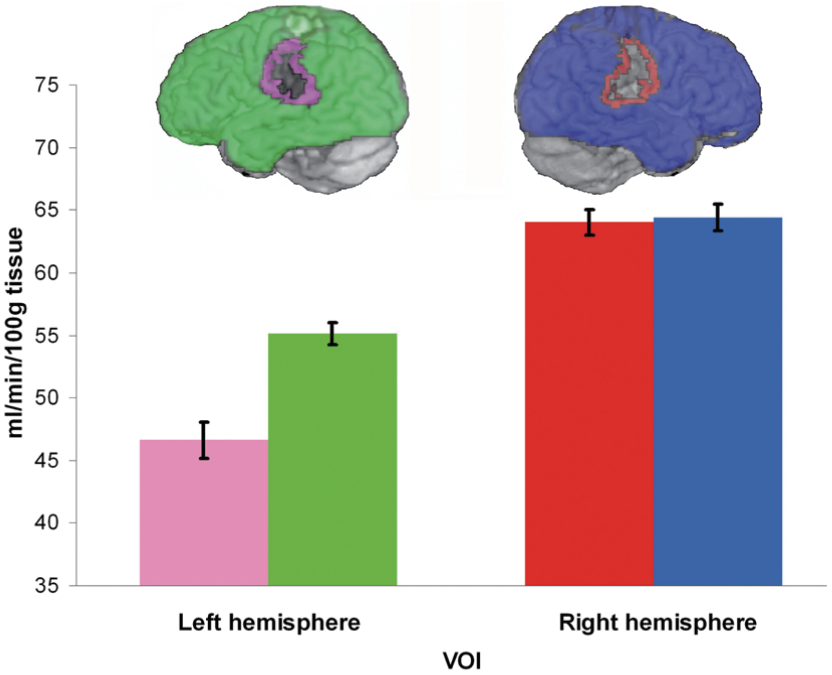

Fig. 1. The image on the left of the inset illustrates the left hemisphere peri-infarct (pink) and remaining (green) masks. The image on the right of the inset illustrates homologous masks (red and blue). The graph depicts results from the volume of interest analysis, in which perfusion values in the peri-infarct and remaining cortex were compared across the two hemispheres. The error bars denote normalized standard error.

tistical Package for the Social Sciences) version 17.0 (Chicago, IL). Perfusion data were subjected to a 2 (left vs. right hemisphere) $\times$ 2 (peri-infarct vs. remaining VOI) within-subjects ANOVA. Planned comparisons included the calculation of Pearson product-moment correlation coefficient to characterize the relationship between perfusion ratios and infarct size, and Spearman rank correlation coefficient to characterize the relationship between perfusion ratios and TPO, a relationship that might not be linear. Statistical significance was defined as $p < 0.05$.

## 3. Results

Cortical infarct size varied from a minimum of 11.31 cc to a maximum of 272.62 cc ($M = 105.91$, *SD* = 76.17). Mean perfusion values were 46.64 (*SD* = 13.37) for LHp and 55.13 (*SD* = 14.97) for LHr (Fig. 1). Higher perfusion values were observed in the right hemisphere, with a mean of 64.01 (*SD* = 16.09) for RHp and 64.43 (*SD* = 15.51) for RHr. The mean LHp/RHp perfusion ratio was 0.7347 (*SD* = 0.1133). The mean LHr/RHr perfusion ratio was 0.8559 (*SD* = 0.0834).

The ANOVA revealed main effects for the factors 'hemisphere', $F(1,16) = 59.7$, $p < 0.001$, $\eta_p^2 = 0.789$, and 'VOI', $F(1,16) = 11.6$, $p = 0.003$, $\eta_p^2 = 0.420$. These main effects were qualified by a significant ordinal interaction between hemisphere and VOI, $F(1,16) = 40.4$, $p < 0.001$, $\eta_p^2 = 0.717$. Thus, left hemisphere values were consistently lower than right hemisphere values, but the magnitude of the difference was greater when comparing peri-infarct values to the remaining hemisphere values. *Post-hoc* pairwise comparisons confirmed that LHp perfusion values were significantly lower than RHp ($t[16] = 8.05$, $p < 0.001$) and RHr ($t[16] = 7.18$, $p < 0.001$) VOIs. In contrast, there was no significant difference between right hemisphere VOIs, $t(16) = 0.35$, $p = 0.73$. A significant difference between values was observed in LHp versus LHr, $t(16) = 5.09$, $p < 0.001$. There was also a significant difference between LHr and right hemisphere VOIs, with LHr demonstrating significantly less perfusion relative to RHp ($t[16] = 4.93$, $p < 0.001$) and RHr ($t[16] = 6.41$, $p < 0.001$) VOIs.

The correlations between TPO and perfusion ratios did not reach statistical significance. Significant negative correlations were revealed between infarct size and both the LHp/RHp perfusion ratio, $r(17) = -0.757$,

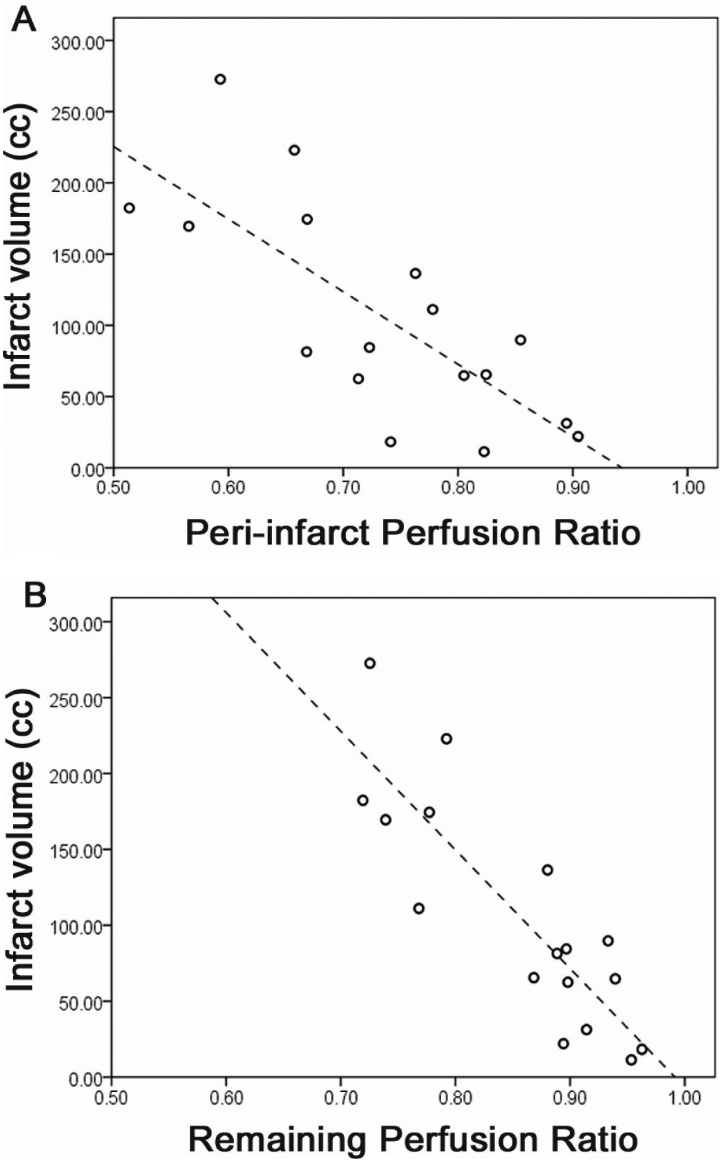

Fig. 2. Scatter plots depicting the relationship between perfusion ratios and infarct volume. The x-axis denotes perfusion ratios; the y-axis represents infarct volume. The dotted lines represent the line of best fit.

$p < 0.001$ (Fig. 2A) and LHr/RHr perfusion ratio $r(17) = -0.8559$, $p < 0.001$ (Fig. 2B). Patients with larger infarcts tended to have lower perfusion ratios.

## 4. Discussion

Whereas a pattern of perfusion deficit resolution is apparent in early stroke recovery [3], our results revealed decreased perfusion in peri-infarct and remaining left hemisphere areas in patients with chronic stroke. Thus, resolution of hypoperfusion may be incomplete in some patients and is not related to TPO. It cannot be assumed that the physiology of remaining structurally intact brain is normal in the chronic stroke population, even as many as 20 years post stroke. Our results also demonstrated that, unlike that seen in early stroke recovery [11,15], infarct size predicted perfusion levels in the chronic phase. Consequently, individuals with larger infarcts may be doubly negatively impacted, since larger infarcts strongly correlated with lower perfusion in the affected hemisphere, and both phenomena are possibly related to poorer clinical status [13,16,22]. Importantly, the potential contributions of peri-infarct tissue to recovery might be compromised by hypoperfusion as there is evidence emphasizing the importance of peri-infarct function for optimal clinical outcomes [10,20].

Much remains to be learned about the relationship between hypoperfusion and function, as there is aforementioned evidence of functional deficits clearly attributed to hypoperfusion that contrasts with evidence of patients with hypoperfusion and infarct to primary language areas who do not demonstrate functional deficits [21]. Information about perfusion deficits and its effects on behaviors may need to be incorporated into future lesion-impairment studies so that a greater degree of accuracy can be achieved when characterizing brain-behavior relationships. It is possible that cerebral perfusion deficits might underlie some of the variability observed in chronic stroke outcomes, and if so, should be accounted for in future studies that relate brain function to stroke recovery.

These results confirm the need for further changes to functional MRI (fMRI) research design in chronic stroke patients since it has been demonstrated that this population often presents with a sustained decrease in the blood oxygen level dependent (BOLD) signal [5, 12]. The majority of stroke recovery studies, however, utilize canonical fMRI protocols that may not account for stroke related changes in blood flow. Based on the present results, future fMRI studies involving chronic stroke patients may be improved by accounting for possible perfusion abnormalities [5]. Furthermore, it is largely unknown how the status of the vessel patency may impact perfusion. It remains to be defined how intra- and extra-cranial stenosis affect perfusion in chronic stroke patients, particularly when other factors such as collateral perfusion [9] may play a significant role.

Our patients constitute a heterogeneous group that is representative of the variety of ischemic stroke etiologies. The mechanism of sustained hypoperfusion in our patients is unknown, and because at least a subset of our population demonstrate hypoperfusion in the absence of stenosis, mechanisms not specific to occlusion should be explored. Stroke-induced changes in neurovascular function, such as cerebrovascular reactivity (CVR), might better explain our results. The co-occurrence of hypoperfusion and reduced CVR has been reported [4,6], and reduced CVR has been reported in peri-infarct regions in both acute and chronic recovery [4,6,8,18], and within chronic recovery in individuals with and without chronic occlusion [8]. Another potential explanation could arise from a supply-demand situation in which the neurons' metabolic needs, and thus perfusion, are reduced because they are not functioning normally within the cortical network. Regardless of the mechanism, it seems hypoperfusion is more common in chronic stroke than previously described. Successful reperfusion treatments have been demonstrated for early recovery; for instance, Hillis and colleagues have shown how reperfusion and subsequent improvement in visual neglect or speech-language abilities can be manipulated by restoring blood flow to hypoperfused areas via pharmacological intervention or surgical procedures [13–15]. We speculate that the chronic patient population might benefit from similar research seeking to improve perfusion in the affected hemisphere, perhaps via intensive treatment [17] or brain stimulation [2], emphasizing the need for further research regarding the mechanism of chronic hypoperfusion.

The peri-infarct mask in the present study was only extended 8 mm beyond the infarct, insinuating that patients might not demonstrate hypoperfusion beyond this region. However, it is possible that at least some patients demonstrate significant perfusion deficits further than 8 mm beyond the infarct. Encouragingly, neurons within hypoperfused areas had not been absorbed into the ischemic core in these patients and may be viable for future treatments that can capitalize on their recruitment. Exploration into the many topics presented in this discussion is currently underway. With the emergence and continued adoption of noninvasive perfusion imaging, it is our hope that cerebral perfusion in chronic stroke and its relationship to functional status will be more thoroughly characterized.

## Acknowledgements

This work was supported by the following grants: DC008355 (PI: JF) and DC009571 (PIs: JF & CR).

We would like to thank: Jiongjiong Wang, PhD, Radiology, University of Pennsylvania, Philadelphia, PA 19104, USA for providing the ASL sequence; Brian M Dale, PhD, Siemens Medical, Morrisville, NC 27560, USA for supporting the ASL sequence under the Master Research Agreement between Siemens Medical and the University of South Carolina; and our patients for their willingness to participate in our research.

## Disclosure/conflict of interest

Paul S. Morgan entered into a Master Research Agreement with Siemens Medical that supports the use of the ASL sequence at the University of South Carolina.
